# Changes in pediatric unintentional injury hospitalizations before and after the COVID-19 pandemic: a retrospective analysis

**DOI:** 10.3389/fpubh.2026.1722038

**Published:** 2026-03-24

**Authors:** Tian Tang, Shaojun Li, Lin Yang, Liang Zhou, Liping Tan

**Affiliations:** 1 Department of Emergency Children’s Hospital of Chongqing Medical University, National Clinical Research Center for Child Health and Disorders, Ministry of Education Key Laboratory of Child Development and Disorders, Chongqing, China; 2 Intelligent Application of Big Data in Pediatrics Engineering Research Center of Chongqing Education Commission of China, Chongqing, China

**Keywords:** COVID-19, epidemiology, hospitalizations, pediatrics, unintentional injury

## Abstract

**Objective:**

This study aimed to analyze the epidemiological characteristics and trends of hospitalizations for unintentional injuries (UI) among children and adolescents (≤18 years) before (2015–2019) and after (2020–2024) the COVID-19 pandemic.

**Methods:**

A retrospective review was conducted on 51,591 UI-related hospital admissions from our university-affiliated children’s hospital. Patients were categorized into five age groups: infants (<1 year), toddlers (1–3 years), preschool-age (4–6 years), school-age (7–12 years), and adolescents (13–18 years). Data were analyzed across the two defined periods.

**Results:**

The total number of UI hospitalizations decreased in Post-COVID compared to pre-COVID. Marked reductions were observed particularly in 2020 and 2022, with a gradual upward trend emerging from 2023 onward—patterns consistent with the evolving impacts of the pandemic. The majority of patients were male and from rural areas throughout the study. However, an increasing yearly trend was observed in the proportion of female patients and those from urban areas after the pandemic. Toddlers consistently had the highest hospitalization rate. A shift in age distribution was noted, with a decreased proportion of cases among infants and toddlers and an increased proportion among school-age children and adolescents post-COVID. Fall/slip were the most common injury mechanism across all ages. Compared with the pre-pandemic period, the proportion of foreign body has decreased significantly in infants, whereas the rate of accidental poisoning has risen markedly among adolescents. Traffic accidents were consistently associated with the highest intensive care unit (ICU) admission rate, the highest readmission rate, and the highest medical costs, yet the lowest rate of recovered/improved. The pattern of body injuries varied with age, with head injuries decreasing and injuries to the extremities, abdomen, and pelvis becoming more common in older age groups. Clinical outcomes improved, with a lower rate of critical illness and higher rates of ICU admission, surgical intervention, and clinical recovered/improved post-COVID.

**Conclusion:**

This study delineates the evolving landscape of pediatric UI before and after the COVID-19 pandemic. While the fundamental causes of injury remain, their proportions and severities have shifted, influenced by changes in children’s living environments and activities. These results underscore the importance of continuous epidemiological surveillance to inform and refine effective, age-specific prevention strategies and to optimize clinical resource allocation for pediatric injury care.

## 1 Introduction

Unintentional injuries (UI) represent a major global public health issue, accounting for up to 90% of all injuries and contributing significantly to morbidity and mortality among children and adolescents (1). Approximately 1 million children succumb to UI every year worldwide. Due to their high associated rates of disability and mortality, UI have attracted widespread attention as a critical public health challenge worldwide (2). Children and adolescents are particularly vulnerable to UI compared to adults (3). The economic burden of pediatric UI is also substantial, encompassing not only direct healthcare costs but also long-term impacts on future income and overall quality of life (4). In China, UI ranks among the top five causes of death, resulting in over 500,000 fatalities annually (5). It remains the leading cause of death for children under 5 years of age, with approximately 26,600 deaths each year—accounting for nearly 15% of all child deaths in this age group (6). Furthermore, disability resulting from UI compounds the problem, as it affects children across more years of life, leading to prolonged personal and societal burdens (7). Despite their high incidence and severe consequences, UI are largely preventable (8). Thus, ongoing monitoring and epidemiological analysis of UI in children are essential for developing and adapting effective prevention and intervention strategies.

Global strategies for pediatric injury prevention have been widely established. The World Health Organization (WHO) has formulated specific plans for child and adolescent injury prevention through the WHO Plan of Action (2006–2015) and the World Report on Child Injury Prevention (8). Similarly, the United States has implemented a national action plan for child injury prevention (9), and the United Kingdom has developed guidelines targeting children under 15 years old (10). These initiatives have demonstrated considerable success in reducing injury rates among children and adolescents. China has also integrated child injury prevention into national policy frameworks, such as the Chinese Child Development Program (2011–2020) and the Healthy China 2030 Plan. Nevertheless, compared to developed nations, China still lacks targeted, clearly-defined, and specific child injury prevention strategies—particularly in its western regions, where the burden of injury remains high (11). Despite extensive research on the causes and impacts of pediatric injuries, significant disparities persist in the distribution of injury risks and access to preventive measures across different socioeconomic groups and geographic areas (12). As the only municipality in western China directly under the central government, Chongqing covers a large area and exhibits a distinct urban–rural divide. It encompasses both metropolitan and mountainous rural settings, each with unique socioeconomic and demographic profiles. These factors may lead to variations in the patterns and types of UI among children compared to other parts of China. Although some studies have examined childhood injury in certain regions, there remains a lack of long-term, systematic surveillance data specific to Chongqing—particularly research spanning major public health events such as the COVID-19 pandemic. The COVID-19 pandemic and associated control measures—including lock downs, stay-at-home orders, and school closures—have profoundly altered children’s daily lives, learning environments, and activity patterns. These changes may have significantly influenced the incidence, nature, and contexts of UI (13). However, empirical evidence regarding these impacts in Chongqing is currently scarce.

This study retrospectively analyzes medical records of children hospitalized for UI between 2015 and 2024—a 10-year period spanning 5 years before and 5 years after the COVID-19 pandemic—at the Children’s Hospital of Chongqing Medical University, a leading pediatric institution and authoritative center in Southwest China. Based on an in-depth review of 51,591 hospitalization cases due to UI, this research systematically examines: (1) changes in trends regarding the types, severity, and outcomes of UI among children pre and post-COVID pandemic; (2) the distribution patterns of such injuries across different age groups (e.g., infants, preschoolers, school-aged children, and adolescents) and their temporal evolution in relation to the pandemic. We hypothesize that social restrictions and environmental changes during the pandemic significantly altered the epidemiological characteristics of childhood UI, potentially leading to an increased proportion of fall/slip and blunt injury, alongside a decrease in traffic-related injuries. The findings will elucidate the dynamic changes in childhood UI in Chongqing over the past decade and quantitatively assess the unique impact of the COVID-19 pandemic as a major public health emergency on injury patterns. These results will not only address a significant regional research gap but also provide scientifically grounded evidence to inform public health authorities, medical institutions, schools, and families. Ultimately, this study aims to support timely adjustments in prevention strategies and optimized resource allocation in the post-pandemic era, thereby enhancing the protection of children from UI and contributing to the achievement of the “Healthy China 2030” strategic goal.

## 2 Materials and methods

### 2.1 Study population

This study employed a retrospective, cross-sectional design using data extracted from the database of the Children’s Hospital Affiliated to Chongqing Medical University between January 2015 and December 2024. In accordance with the methodology employed by Lee et al. (14–16), the present study designated the periods from January 2015 to December 2019 (pre-COVID) and January 2020 to December 2024 (post-COVID) to investigate changes in pediatric UI hospitalizations during the COVID-19 outbreak. This analytical approach, which utilizes multi-year aggregated data in preference to single-year estimates, confers a more stable data foundation and greater statistical power, thereby facilitating a more reliable evaluation of the overarching trends characterizing this epoch. The hospital is situated in Yuzhong District, a central urban area in southwestern Chongqing, and serves as the largest national tertiary comprehensive children’s hospital in Western China.

A total of 51,591 hospitalized children and adolescents (aged ≤18 years) with UI were included in the review. Participants were categorized into the following age groups: infants (<1 year), toddlers (1–3 years), preschool-age children (4–6 years), school-age children (7–12 years), and adolescents (13–18 years). The study involving human participants were reviewed and approved by the Clinical Research Management Committee of Children’s Hospital Affiliated to Chongqing Medical University (IRB No. 2025-503).

### 2.2 Measures

The demographic variables considered in this study included age, sex, type of injury and injury site. To assess injury severity, several clinical outcome indicators were incorporated, including type of admission (e.g., critical, severe, general), admission to the ICU, surgical interventions, and readmission. Additional outcomes analyzed were prognosis (categorized as recovered/improved, not improved, died, or others), length of hospital stay and total hospital costs.

UI were classified according to the International Classification of Diseases, 10th Revision (ICD-10), under code range V01-X59. Specific injury types included: thermal injury (X00-X19), fall/slip (W00-W19), blunt injury (W20-W24), accidental poisoning (X40-X49), foreign body (W44, W75-W84), drowning (W65-W74), traffic accidents (V01-V99), sharp object injury (W25-W31), animal-related injury (W50-W64), and other accidental injuries (W32-W43, W45-W49, W85-W99, X20-X39, X50-X59).

Based on ICD-10 codes, the anatomical sites of injury were categorized as follows: head (S00-S09), neck (S10-S19), thorax (S20-S29), abdomen and pelvis—including lower back and genitals (S30-S39), shoulders and upper arms (S40-S49), elbows and forearms (S50-S59), wrists and hands (S60-S69), hips and thighs (S70-S79), knees and lower legs (S80-S89), and ankles and feet (S90-S99).

### 2.3 Statistical analysis

Statistical analyses were performed using SPSS 19.0 (IBM, Chicago, IL, USA) and R software (version 4.0.3; R Foundation for Statistical Computing, Vienna, Austria). Graphical representations were generated with Prism 8.4.2 (GraphPad, San Diego, CA, USA). Continuous variables that did not follow a normal distribution are expressed as median and interquartile range (IQR). Categorical variables are summarized as frequencies and percentages (*n* [%]). Group comparisons were conducted using the Mann–Whitney U test for non-normally distributed continuous data and the Chi-squared test for categorical variables. To evaluate the impact of the COVID-19 pandemic on pediatric UI, an interrupted time series (ITS) analysis was employed as the primary analytical approach. *p* < 0.05 was considered statistically significant for all tests.

## 3 Results

### 3.1 Characteristics and demographics of pediatric injury

**Figure 1:**
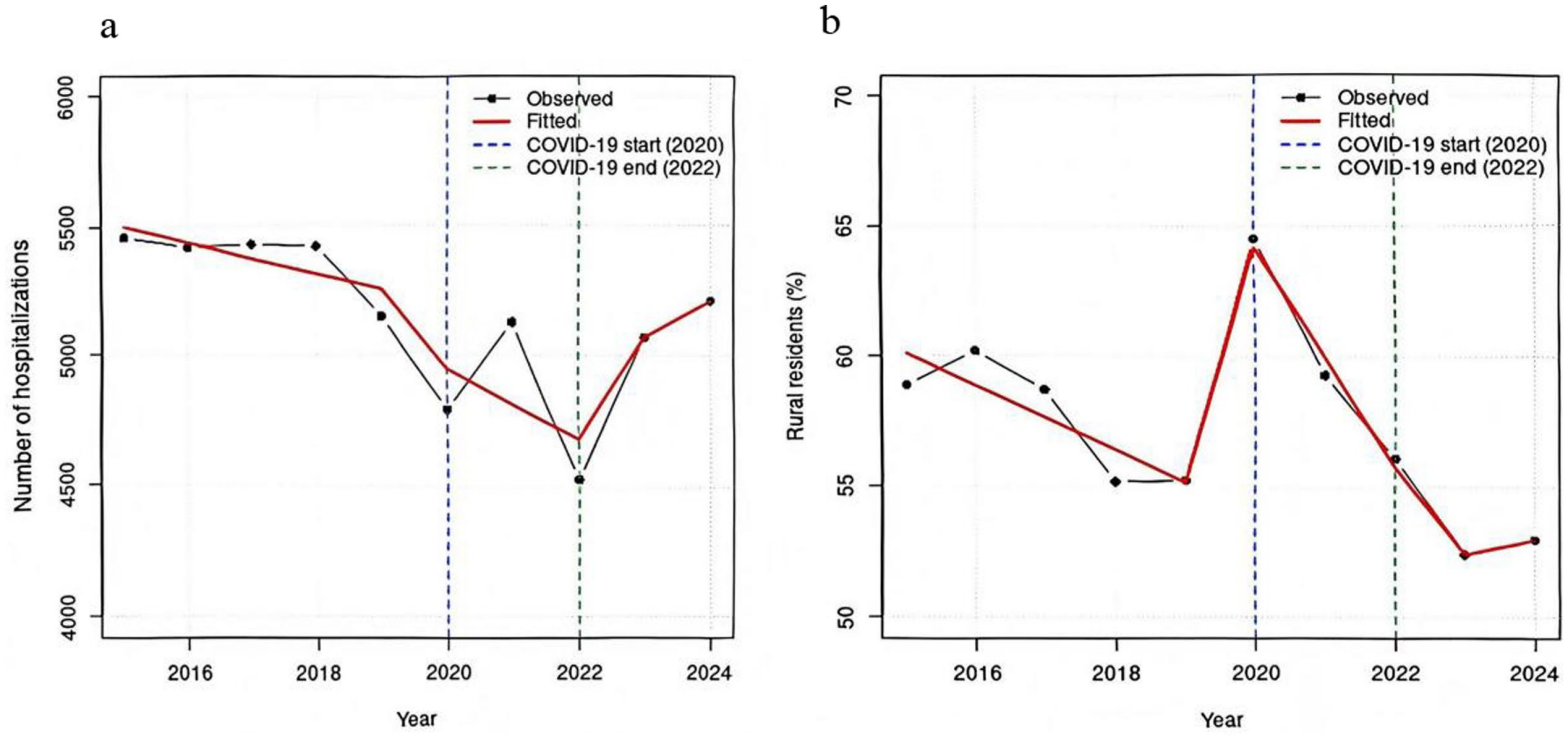
Interrupted time series analysis of pediatric UI hospitalizations, 2015–2024, showing: **(a)** Number of hospitalizations; **(b)** Rural proportion.

This study retrospectively analyzed data from children hospitalized due to UI over a 10-year period, including a total of 51,591 patients—26,865 from the pre-COVID-19 period and 24,726 from the post-COVID-19 period. Prior to the pandemic, the number of pediatric hospitalizations for UI remained generally stable, with a slight declining trend. Following the onset of COVID-19, a marked decline was observed in 2020. Although there was a modest rebound in 2021, admissions reached their nadir in 2022. Subsequently, from 2023 onward, hospitalization numbers gradually recovered to levels approaching those seen in the pre-pandemic period. Patients were stratified into age groups as follows: infants (*n* = 3,393), toddlers (*n* = 18,969), preschoolers (*n* = 12,837), school-aged children (*n* = 13,720), and adolescents (*n* = 2,672). Results indicated that males constituted the majority of hospitalizations for UI (63.3%), with the male proportion increasing significantly with age. However, following the onset of the COVID-19 pandemic, the proportion of hospitalized male children decreased, while that of females increased. Children from rural areas account for the majority of pediatric hospitalizations due to UI. During the pre-pandemic period, the proportion of hospitalized pediatric UI patients from rural areas showed a gradual decline. However, in the immediate aftermath of the COVID-19 outbreak in 2020, this proportion increased markedly, reaching its highest point in the decade. Thereafter, it began to decrease slowly, eventually falling to its lowest level during the late-pandemic period of 2023–2024 (Tables 1, 2, 3; Figures 1a,b).

**Table 1:** General characteristics of pediatric injury.

Variable	Pre-COVID (2015–2019)	Post-COVID (2020–2024)	Total (2015–2024)	*p*
	*N* = 26,865	*N* = 24,726	*N* = 51,591	
Gender				0.039
Male	17,128 (63.8%)	15,547 (62.9%)	32,675 (63.3%)	
Female	9,737 (36.2%)	9,179 (37.1%)	18,916 (36.7%)	
Residence				<0.001
Urban	11,366 (42.3%)	10,954 (44.3%)	22,320 (43.3%)	
Rural	15,499 (57.7%)	13,772 (55.7%)	29,271 (56.7%)	
Age (group), years				<0.001
Infants (0–1)	2016 (7.5%)	1,377 (5.6%)	3,393 (6.6%)	
Toddlers (1–3)	10,712 (39.9%)	8,257 (33.4%)	18,969 (36.7%)	
Preschool-age children (4–6)	6,453 (24%)	6,384 (25.8%)	12,837 (24.9%)	
School-age children (7–12)	6,633 (24.7%)	7,087 (28.7%)	13,720 (26.6%)	
Adolescents (13–18)	1,051 (3.9%)	1,621 (6.6%)	2,672 (5.2%)	
Season				<0.001
Spring	7,200 (26.8%)	6,821 (27.6%)	14,021 (27.2%)	
Summer	7,335 (27.3%)	6,654 (26.9%)	13,989 (27.1%)	
Autumn	6,495 (24.2%)	6,202 (25.1%)	12,697 (24.6%)	
Winter	5,835 (21.7%)	5,049 (20.4%)	10,884 (21.1%)	
Patient condition				<0.001
Critical	4,188 (15.6%)	3,158 (12.8%)	7,346 (14.2%)	
Severity	4,596 (17.1%)	4,638 (18.8%)	9,234 (17.9%)	
General	18,081 (67.3%)	16,930 (68.5%)	35,011 (67.9%)	
Mode				<0.001
Thermal injury	2027 (7.5%)	1745 (7.1%)	3,772 (7.3%)	
Fall, slip	12,536 (46.7%)	12,272 (49.6%)	24,808 (48.1%)	
Blunt injury	1733 (6.5%)	1784 (7.2%)	3,517 (6.8%)	
Foreign body	3,596 (13.4%)	2,629 (10.6%)	6,225 (12.1%)	
Accidental poisoning	783 (2.9%)	740 (3%)	1,523 (3%)	
Drowning	102 (0.4%)	128 (0.5%)	230 (0.4%)	
Traffic accident	3,600 (13.4%)	3,110 (12.6%)	6,710 (13%)	
Sharp injury	652 (2.4%)	774 (3.1%)	1,426 (2.8%)	
Animal injury	453 (1.7%)	484 (2%)	937 (1.8%)	
Others and unknown	1,383 (5.1%)	1,060 (4.3%)	2,443 (4.7%)	
ICU				<0.001
Yes	765 (2.8%)	1,134 (4.6%)	1899 (3.7%)	
No	26,100 (97.2%)	23,592 (95.4%)	49,692 (96.3%)	
Operation				<0.001
Yes	16,038 (59.7%)	15,706 (63.5%)	31,744 (61.5%)	
No	10,827 (40.3%)	9,020 (36.5%)	19,847 (38.5%)	
Prognosis				<0.001
Recovered/improved	25,642 (95.5%)	23,999 (97.1%)	49,641 (96.2%)	
Not improved/ died/others	1,223 (4.5%)	727 (2.9%)	1950 (3.8%)	
Readmission				<0.001
Yes	814 (3%)	301 (1.2%)	1,115 (2.2%)	
No	26,051 (97%)	24,425 (98.8%)	50,476 (97.8%)	
Length of hospital stay	7 (4–10)	7 (4–9)	7(4–10)	<0.001
Total hospital costs	9060.5 (5057–15545.4)	9467.9 (5451.6–13723.9)	9287.1 (5213.5–14,628)	0.525

**Table 2:** General characteristics of pediatric injury patients by age group.

Characteristic	Pre-COVID (2015–2019)					*p*-value	Post-COVID (2020–2024)					*p*-value
	Infants	Toddlers	Preschool-age children	School-age children	Adolescents		Infants	Toddlers	Preschool-age children	School-age children	Adolescents	
	*n* = 2016	*n* = 10,712	*n* = 6,453	*n* = 6,633	*n* = 1,051		*n* = 1,377	*n* = 8,257	*n* = 6,384	*n* = 7,087	*n* = 1,621	
Gender						<0.001						<0.001
Male	1,232 (61.1%)	6,444 (60.2%)	4,057 (62.9%)	4,550 (68.6%)	845 (80.4%)		776 (56.4%)	4,916 (59.5%)	4,011 (62.8%)	4,681 (66.1%)	1,163 (71.7%)	
Female	784 (38.9%)	4,268 (39.8%)	2,396 (37.1%)	2083 (31.4%)	206 (19.6%)		601 (43.6%)	3,341 (40.5%)	2,373 (37.2%)	2,406 (33.9%)	458 (28.3%)	
Residence						0.02						<0.001
Urban	893 (44.3%)	4,560 (42.6%)	2,789 (43.2%)	2,722 (41%)	402 (38.2%)		551 (40%)	3,398 (41.2%)	3,005 (47.1%)	3,293 (46.5%)	707 (43.6%)	
Rural	1,123 (55.7%)	6,152 (57.4%)	3,664 (56.8%)	3,911 (59%)	649 (61.8%)		826 (60%)	4,859 (58.8%)	3,379 (52.9%)	3,794 (53.5%)	914 (56.4%)	
Patient condition						<0.001						<0.001
Critical	570 (28.3%)	1,452 (13.6%)	1,048 (16.2%)	977 (14.7%)	141 (13.4%)		304 (22.1%)	1,253 (15.2%)	650 (10.2%)	760 (10.7%)	191 (11.8%)	
Severity	605 (30%)	2,507 (23.4%)	740 (11.5%)	663 (10%)	81 (7.7%)		613 (44.5%)	1901 (23%)	1,063 (16.7%)	932 (13.2%)	129 (8%)	
General	841 (41.7%)	6,753 (63%)	4,665 (72.3%)	4,993 (75.3%)	829 (78.9%)		450 (33.4%)	5,103 (61.8%)	4,671 (73.2%)	5,395 (76.1%)	1,301 (80.3%)	
Mode						<0.001						<0.001
Thermal injury	352 (17.5%)	1,410 (13.2%)	178 (2.8%)	85 (1.3%)	2 (0.2%)		244 (17.7%)	1,181 (14.3%)	211 (3.3%)	106 (1.5%)	3 (0.2%)	
Fall, slip	850 (42.2%)	3,804 (35.5%)	3,450 (53.5%)	3,754 (56.6%)	678 (64.5%)		643 (46.7%)	3,014 (36.5%)	3,728 (58.4%)	3,975 (56.1%)	912 (56.3%)	
Blunt injury	80 (4%)	789 (7.4%)	460 (7.1%)	366 (5.5%)	38 (3.6%)		49 (3.6%)	689 (8.3%)	493 (7.7%)	493 (7%)	60 (3.7%)	
Foreign body	381 (18.9%)	2,629 (24.5%)	305 (4.7%)	264 (4%)	17 (1.6%)		197 (14.3%)	1828 (22.1%)	299 (4.7%)	282 (4%)	23 (1.4%)	
Accidental poisoning	48 (2.4%)	353 (3.3%)	150 (2.3%)	169 (2.5%)	63 (6%)		52 (3.8%)	266 (3.2%)	61 (1%)	169 (2.4%)	192 (11.8%)	
Drowning	3 (0.1%)	50 (0.5%)	20 (0.3%)	29 (0.4%)	0		4 (0.3%)	63 (0.8%)	23 (0.4%)	36 (0.5%)	2 (0.1%)	
Traffic accident	155 (7.7%)	997 (9.3%)	1,203 (18.6%)	1,116 (16.8%)	129 (12.3%)		117 (8.5%)	669 (8.1%)	1,004 (15.7%)	1,135 (16%)	185 (11.4%)	
Sharp injury	14 (0.7%)	327 (3.1%)	146 (2.3%)	148 (2.2%)	17 (1.6%)		11 (0.8%)	305 (3.7%)	214 (3.4%)	216 (3%)	28 (1.7%)	
Animal injury	15 (0.7%)	102 (1%)	115 (1.8%)	185 (2.8%)	36 (3.4%)		6 (0.4%)	97 (1.2%)	108 (1.7%)	215 (3%)	58 (3.6%)	
Others and unknown	118 (5.9%)	251 (2.3%)	426 (6.6%)	517 (7.8%)	71 (6.8%)		54 (3.9%)	145 (1.8%)	243 (3.8%)	460 (6.5%)	158 (9.7%)	

**Table 3:** Characteristics of participants by year of admission.

Variable	2015	2016	2017	2018	2019	2020	2021	2022	2023	2024	*p*
	*n* = 5,460	*n* = 5,418	*n* = 5,427	*n* = 5,415	*n* = 5,145	*n* = 4,792	*n* = 5,123	*n* = 4,529	*n* = 5,071	*n* = 5,211	
Gender											0.014
Male	3,553 (65.1%)	3,485 (64.3%)	3,454 (63.6%)	3,437 (63.5%)	3,199 (62.2%)	2,950 (61.6%)	3,224 (62.9%)	2,898 (64%)	3,187 (62.8%)	3,288 (63.1%)	
Female	1907 (34.9%)	1933 (35.7%)	1973 (36.4%)	1978 (36.5%)	1946 (37.8%)	1842 (38.4%)	1899 (37.1%)	1,631 (36%)	1884 (37.2%)	1923 (36.9%)	
Residence											<0.001
Urban	2,243 (41.1%)	2,157 (39.8%)	2,241 (41.3%)	2,426 (44.8%)	2,299 (44.7%)	1702 (35.5%)	2087 (40.7%)	1988 (43.9%)	2,415 (47.6%)	2,449 (47%)	
Rural	3,217 (58.9%)	3,261 (60.2%)	3,186 (58.7%)	2,989 (55.2%)	2,846 (55.3%)	3,090 (64.5%)	3,036 (59.3%)	2,541 (56.1%)	2,656 (52.4%)	2,762 (53%)	
Age (group), years											<0.001
Infants (0–1)	405 (7.4%)	442 (8.2%)	428 (7.9%)	390 (7.2%)	351 (6.8%)	298 (6.2%)	337 (6.6%)	223 (4.9%)	265 (5.2%)	254 (4.9%)	
Toddlers (1–3)	2,121 (38.8%)	2,127 (39.3%)	2,125 (39.2%)	2,183 (40.3%)	2,156 (41.9%)	2027 (42.3%)	1834 (35.8%)	1,492 (32.9%)	1,493 (29.4%)	1,411 (27.1%)	
Preschool-age children (4–6)	1,374 (25.2%)	1,313 (24.2%)	1,341 (24.7%)	1,238 (22.9%)	1,187 (23.1%)	1,159 (24.2%)	1,320 (25.8%)	1,198 (26.5%)	1,337 (26.4%)	1,370 (26.3%)	
School-age children (7–12)	1,374 (25.2%)	1,342 (24.8%)	1,335 (24.6%)	1,355 (25%)	1,227 (23.8%)	1,095 (22.9%)	1,336 (26.1%)	1,327 (29.3%)	1,600 (31.6%)	1729 (33.2%)	
Adolescents (13–18)	186 (3.4%)	194 (3.6%)	198 (3.6%)	249 (4.6%)	224 (4.4%)	213 (4.4%)	296 (5.8%)	289 (6.4%)	376 (7.4%)	447 (8.6%)	
Season											<0.001
Spring	1,465 (26.8%)	1,394 (25.7%)	1,410 (26%)	1,451 (26.8%)	1,480 (28.8%)	1,350 (28.2%)	1,366 (26.7%)	1,300 (28.7%)	1,379 (27.2%)	1,426 (27.4%)	
Summer	1,418 (26%)	1,452 (26.8%)	1,504 (27.7%)	1,443 (26.6%)	1,518 (29.5%)	1,327 (27.7%)	1,402 (27.3%)	1,260 (27.8%)	1,307 (25.8%)	1,358 (26.1%)	
Autumn	1,326 (24.3%)	1,373 (25.3%)	1,360 (25.1%)	1,333 (24.6%)	1,103 (21.4%)	1,231 (25.7%)	1,253 (24.5%)	1,058 (23.4%)	1,273 (25.1%)	1,387 (26.6%)	
Winter	1,251 (22.9%)	1,199 (22.1%)	1,153 (21.2%)	1,188 (21.9%)	1,044 (20.3%)	884 (18.4%)	1,102 (21.5%)	911 (20.1%)	1,112 (21.9%)	1,040 (20%)	
Patient condition											<0.001
Critical	1,102 (20.2%)	978 (18.1%)	728 (13.4%)	670 (12.4%)	710 (13.8%)	687 (14.3%)	769 (15%)	499 (11%)	599 (11.8%)	604 (11.6%)	
Severity	715 (13.1%)	823 (15.2%)	993 (18.3%)	1,047 (19.3%)	1,018 (19.8%)	984 (20.5%)	932 (18.2%)	886 (19.6%)	948 (18.7%)	888 (17%)	
General	3,643 (66.7%)	3,617 (66.8%)	3,706 (68.3%)	3,698 (68.3%)	3,417 (66.4%)	3,121 (65.1%)	3,422 (66.8%)	3,144 (69.4%)	3,524 (69.5%)	3,719 (71.4%)	
Mode											<0.001
Thermal injury	411 (7.5%)	410 (7.6%)	429 (7.9%)	370 (6.8%)	407 (8%)	379 (7.9%)	409 (8%)	321 (7.1%)	335 (6.6%)	301 (5.8%)	
Fall, slip	2,445 (44.8%)	2,368 (43.7%)	2,694 (49.6%)	2,587 (47.8%)	2,442 (47.5%)	2,240 (46.7%)	2,592 (50.6%)	2,248 (49.6%)	2,500 (49.3%)	2,692 (51.7%)	
Blunt injury	347 (6.4%)	286 (5.3%)	317 (5.8%)	359 (6.6%)	424 (8.2%)	310 (6.5%)	340 (6.6%)	350 (7.8%)	377 (7.4%)	407 (7.8%)	
Foreign body	649 (11.9%)	722 (13.3%)	697 (12.8%)	763 (14.1%)	765 (14.9%)	747 (15.6%)	542 (10.6%)	450 (9.9%)	476 (9.4%)	414 (7.9%)	
Accidental poisoning	214 (3.9%)	176 (3.2%)	138 (2.5%)	132 (2.4%)	123 (2.4%)	137 (2.9%)	112 (2.2%)	125 (2.8%)	179 (3.5%)	187 (3.6%)	
Drowning	23 (0.4%)	25 (0.5%)	18 (0.3%)	19 (0.4%)	17 (0.3%)	18 (0.4%)	29 (0.6%)	26 (0.6%)	29 (0.6%)	26 (0.5%)	
Traffic accident	811 (14.9%)	791 (14.6%)	776 (14.3%)	650 (12%)	572 (11.1%)	567 (11.8%)	599 (11.7%)	571 (12.6%)	665 (13.1%)	708 (13.6%)	
Sharp injury	103 (1.9%)	114 (2.1%)	139 (2.6%)	172 (3.2%)	124 (2.4%)	163 (3.4%)	153 (3%)	131 (2.9%)	152 (3%)	175 (3.4%)	
Animal injury	90 (1.6%)	82 (1.5%)	75 (1.4%)	109 (2%)	97 (1.9%)	56 (1.2%)	99 (2%)	93 (2.1%)	129 (2.5%)	107 (2.1%)	
Others and unknown	367 (6.7%)	444 (8.2%)	144 (2.7%)	254 (4.7%)	174 (3.4%)	175 (3.7%)	248 (4.8%)	214 (4.7%)	229 (4.5%)	194 (3.7%)	
ICU											<0.001
Yes	210 (3.8%)	194 (3.6%)	143 (2.6%)	107 (2%)	111 (2.2%)	177 (3.7%)	208 (4.1%)	222 (4.9%)	274 (5.4%)	253 (4.9%)	
No	5,250 (96.2%)	5,224 (96.4%)	5,284 (97.4%)	5,308 (98%)	5,034 (97.8%)	4,615 (96.3%)	4,915 (95.9%)	4,307 (95.1%)	4,797 (94.6%)	4,958 (95.1%)	
Operation											<0.001
Yes	3,092 (56.6%)	3,085 (56.9%)	3,248 (59.8%)	3,435 (63.4%)	3,178 (61.8%)	2,966 (61.9%)	3,199 (62.4%)	2,833 (62.6%)	3,216 (63.4%)	3,393 (65.1%)	
No	2,368 (43.4%)	2,333 (43.1%)	2,179 (40.2%)	1980 (36.6%)	1967 (38.2%)	1826 (38.1%)	1924 (37.6%)	1,696 (37.4%)	1855 (36.6%)	1818 (34.9%)	
Prognosis											<0.001
Recovered/improved	4,797 (87.9%)	4,748 (87.6%)	4,929 (90.8%)	4,962 (91.6%)	4,653 (90.4%)	4,638 (96.8%)	4,962 (96.9%)	4,397 (97.1%)	4,917 (97%)	5,085 (97.6%)	
Not improved/died/others	663 (12.1%)	670 (12.4%)	498 (9.2%)	453 (8.4%)	492 (9.6%)	154 (3.2%)	161 (3.1%)	132 (2.9%)	154 (3%)	126 (2.4%)	

**Figure 2:**
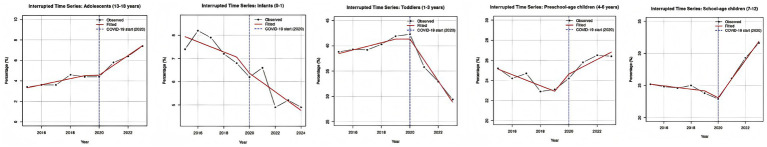
Interrupted time series analysis of pediatric UI hospitalizations, 2015–2024, showing: age distribution.

UI were more frequent during spring and summer. The proportion of critical and severity ill children decreased after the pandemic compared to the pre-COVID-19 period, with a consistent declining trend observed across advancing age groups. Regarding age-specific distribution, toddlers had the highest hospitalization rate, followed by school-aged children, preschoolers, infants, and adolescents. Furthermore, the age distribution of pediatric UI hospitalizations shifted toward older children following the pandemic onset. Specifically, the proportion of hospitalized infants and toddlers decreased significantly after the COVID-19 outbreak, whereas the proportion of school-aged children and adolescents increased markedly. The proportion of preschool-aged children showed a slight increase, though the change was not substantial (Tables 1, 2, 3; Figure 2).

### 3.2 Mechanisms of pediatric injuries

**Figure 3:**
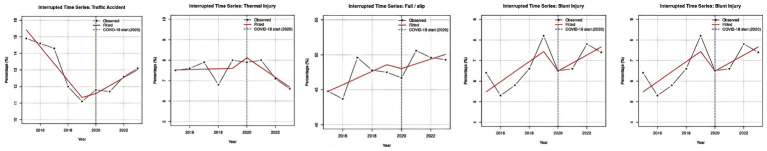
Interrupted time series analysis of pediatric UI hospitalizations, 2015–2024, showing distribution of five common injury types.

Among patients hospitalized due to UI, the five most common types are fall/slip, traffic accident, foreign body, thermal injuries, and blunt injury. Following the pandemic, the proportion of hospitalizations due to fall/slip increased significantly, while those due to foreign body decreased markedly. Proportions for blunt injury showed a slight, non-significant increase, and both thermal injury and traffic accident demonstrated a generally stable but slightly declining trend. Fall/slip represent the most frequent type of UI across all age groups. Among infants and toddlers, foreign body and thermal injury are also common. Preschool and school-aged children are most often affected by traffic accident and blunt injury, while adolescents most frequently experience traffic accident and poisoning. Compared with the pre-pandemic period, the proportion of foreign body has decreased significantly in infants, whereas the rate of accidental poisoning has risen markedly among adolescents (Tables 1, 2, 3; Figure 3).

### 3.3 Anatomical site of injury

**Figure 4:**
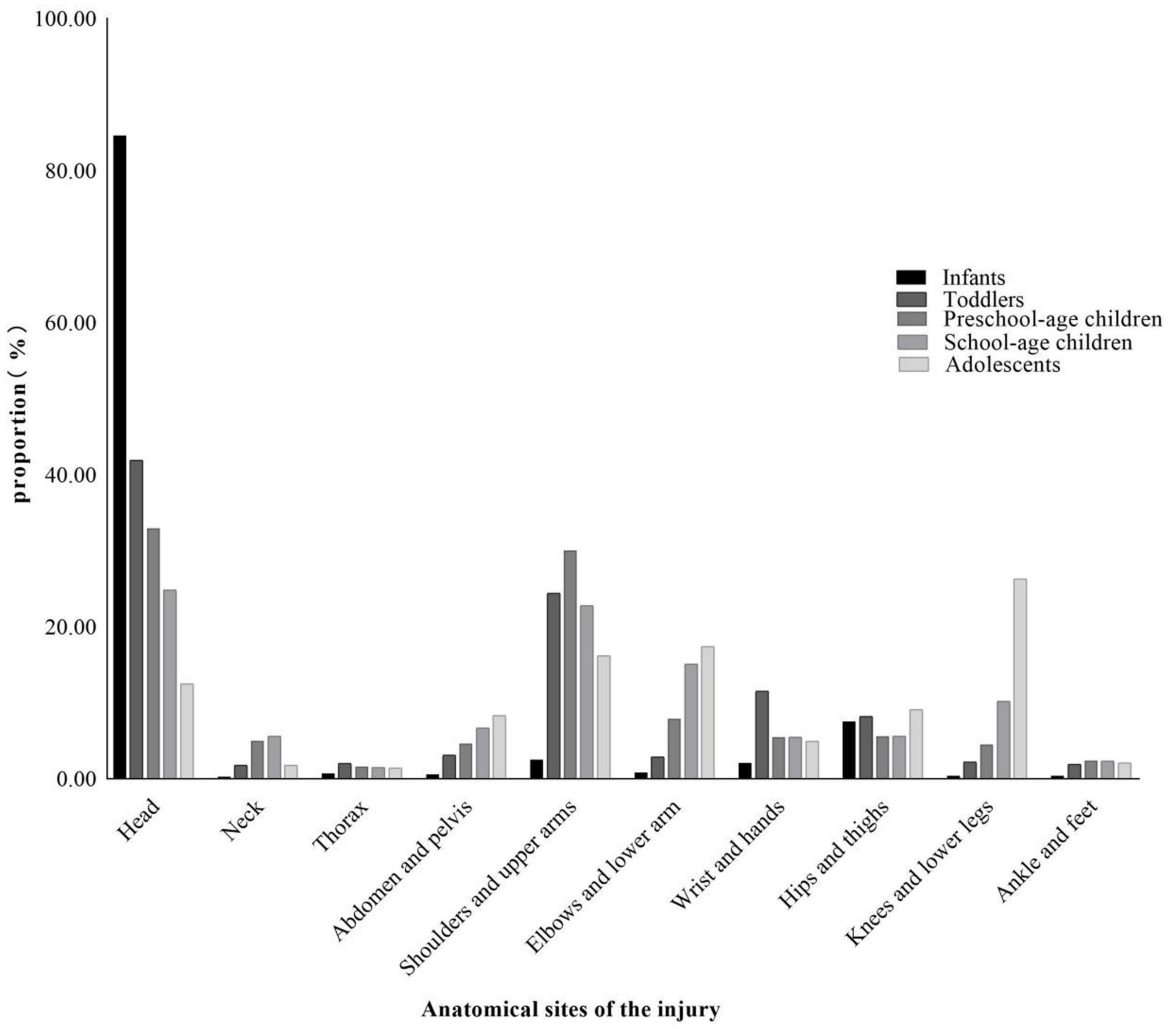
Anatomical site of injury according to age group.

Across all age groups except teenagers, the most frequently injured body site is the head, with incidence rates highest in infants (84.6%), followed by toddlers (41.9%), preschool children (32.9%), and school-aged children (24.8%). In contrast, the most common injury sites among teenagers are the knees and lower legs, accounting for 26.3% of cases. Overall, the data showed a clear trend: younger children are more prone to head injuries, as well as injuries to the abdomen and pelvis, while injuries to the elbows and lower arm, knees and lower legs become more common with increasing age (Figure 4).

### 3.4 Medical attention for pediatric injury

**Figure 5:**
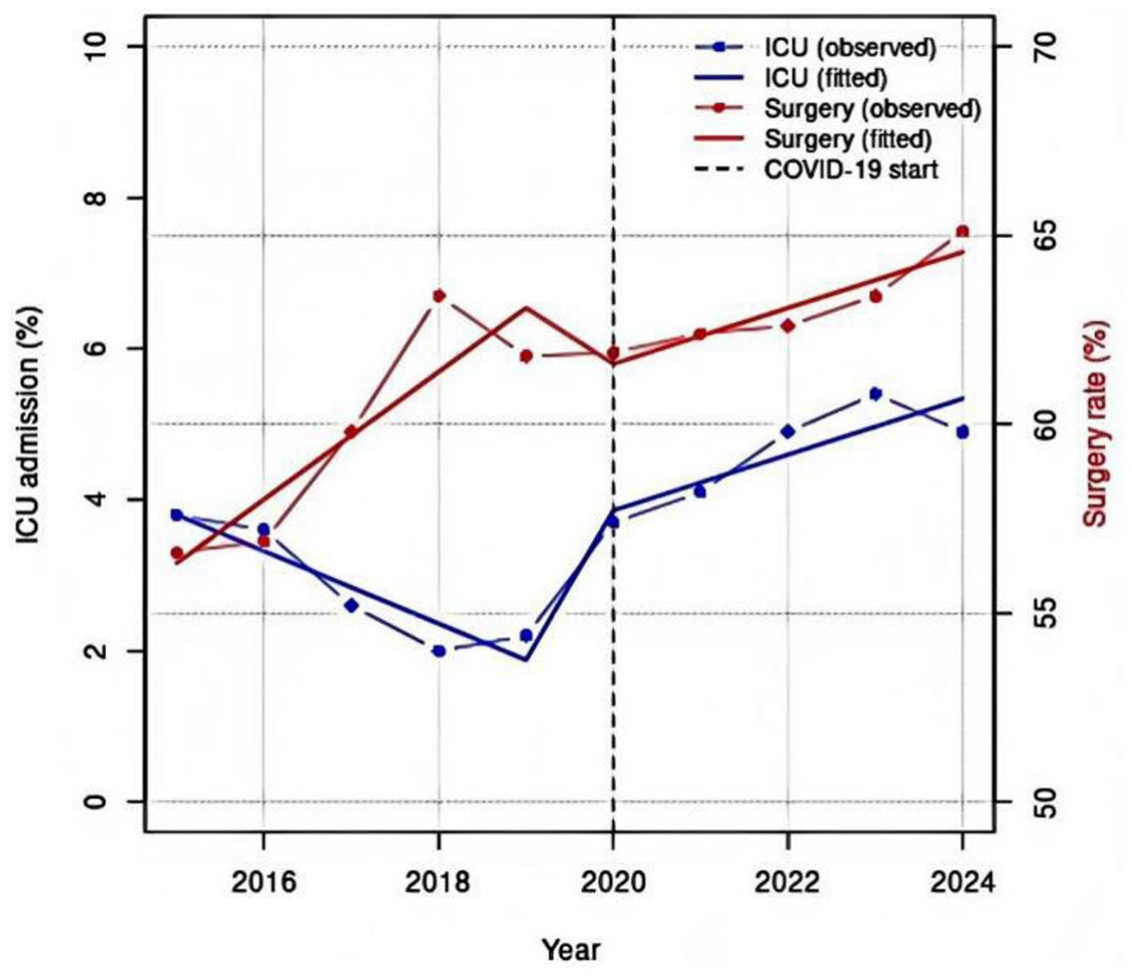
Interrupted time series analysis of pediatric UI hospitalizations, 2015–2024, showing: Surgery rate and ICU admission rate.

Prior to the pandemic, the ICU admission rate for pediatric UI showed a declining trend. In the post-pandemic period, however, this rate rebounded sharply and remained significantly higher than pre-pandemic levels. In contrast, the rate of surgical interventions exhibited a gradual increasing trend throughout the entire study period; although transient fluctuations occurred following the pandemic onset, they did not alter the underlying upward trajectory. Following the pandemic, rates of recovered/improved among children hospitalized for UI were higher compared to the pre-COVID era, while readmission rates and total length of hospital stay decreased. Hospitalization costs showed no significant changes. However, the study also indicated that both the duration of hospital stay and medical expenses tended to increase with advancing age groups (Table 1, 3; Figure 5).

**Table 4:** Outcomes of pediatric injury patients by age group.

	Pre-COVID (2015–2019)						Post-COVID (2020–2024)					
Outcome	Infants	Toddlers	Preschool-age children	School-age children	Adolescents	*p*-value	Infants	Toddlers	Preschool-age children	School-age children	Adolescents	*p*-value
	*n* = 2016	*n* = 10,712	*n* = 6,453	*n* = 6,633	*n* = 1,051		*n* = 1,377	*n* = 8,257	*n* = 6,384	*n* = 7,087	*n* = 1,621	
ICU						<0.001						<0.001
Yes	67 (3.3%)	263 (2.5%)	173 (2.7%)	237 (3.6%)	25 (2.4%)		109 (7.9%)	368 (4.5%)	239 (3.7%)	322 (4.5%)	96 (6%)	
No	1949 (96.7%)	10,449 (97.5%)	6,280 (97.3%)	6,396 (96.4%)	1,026 (97.6%)		1,268 (92.1%)	7,889 (95.5%)	6,145 (96.3%)	6,765 (95.5%)	1,525 (94%)	
Operation						<0.001						<0.001
Yes	745 (37%)	6,300(58.8%)	3,953 (61.3%)	4,322 (65.2%)	718 (68.3%)		440 (32%)	4,876 (59.1%)	4,293 (67.2%)	4,906 (69.2%)	1,092 (67.4%)	
No	1,271 (63%)	4,412(41.2%)	2,500 (38.7%)	2,311 (34.8%)	333 (31.7%)		937 (68%)	3,381 (40.9%)	2091 (32.8%)	2,181 (30.8%)	529 (32.6%)	
Prognosis						<0.001						<0.001
Recovered/improved	1930 (95.7%)	10,357 (96.7%)	6,161 (95.5%)	6,255 (94.3%)	939 (89.3%)		1,307 (94.9%)	8,011 (97%)	6,252 (97.9%)	6,887 (97.2%)	1,542 (95.1%)	
Not improved/died/others	86 (4.3%)	355 (3.3%)	292 (4.5%)	378 (5.7%)	112 (10.7%)		70 (5.1%)	246 (3%)	132 (2.1%)	200 (2.8%)	79 (4.9%)	
Readmission						<0.001						0.005
Yes	28 (1.4%)	226 (2.1%)	218 (3.4%)	267 (4%)	75 (7.1%)		13 (0.9%)	93 (1.1%)	59 (1%)	108 (1.5%)	28 (1.7%)	
No	1988 (98.6%)	10,486 (97.9%)	6,235 (96.6%)	6,366 (96%)	976 (92.9%)		1,364 (99.1%)	8,164 (98.9%)	6,325 (99%)	6,979 (98.5%)	1,593 (98.3%)	
Length of hospital stay	6 (4–10)	6 (4–9)	7 (5–10)	8 (5–10)	8.5 (6–11)	<0.001	6 (4–10)	6 (4–9)	7 (5–9)	7 (5–9)	8 (5–10)	<0.001
Total hospital costs	6983.2 (4592.6–12286.3)	7208.2 (4831.2–12,812)	10935.7 (5450.1–16343.1)	12146.6 (5908–17670.6)	14180.6 (7340.2–21015.1)	<0.001	2213.3 (1382.1–3730.2)	7736.4 (4933.2–12120.6)	10484.1 (5997.5–13920.5)	10941.8 (6263.6–14661.6)	11917.9 (6605.1–17074.6)	**<0.001**

### 3.5 Clinical results of common injuries

During the COVID-19 pandemic, the five most common causes of pediatric hospitalization due to UI remained consistent both before and after the outbreak: fall/slip (*n* = 12,536 vs. 12,272), traffic accident (*n* = 3,600 vs. 3,110), foreign body (*n* = 3,596 vs. 2,629), thermal injury (*n* = 2,027 vs. 1,745), and blunt injury (*n* = 1,733 vs. 1,784). Among these, patients with traffic accident exhibited the highest ICU admission rate, the highest readmission rate, and the highest medical costs, yet the lowest rate of recovered/improved. In contrast, cases involving foreign body injuries required surgical intervention most frequently, but were associated with the shortest length of hospital stay and the lowest medical expenses (Table 5).

**Table 5:** Outcomes of pediatric main injury.

Outcome	Pre-COVID-19 (2015–2019)					*p*-value	Post-COVID-19 (2020–2024)					*p*-value
	Thermal injury	Fall, slip	Blunt injury	Foreign body	Traffic accident		Thermal injury	Fall, slip	Blunt injury	Foreign body	Traffic accident	
	*n* = 2027	*n* = 12,536	*n* = 1733	*n* = 3,596	*n* = 3,600		*n* = 1745	*n* = 12,272	*n* = 1784	*n* = 2,629	*n* = 3,110	
ICU						<0.001						<0.001
Yes	13 (0.6%)	265 (2.1%)	29 (1.7%)	78 (2.2%)	257 (7.1%)		26 (1.5%)	376 (3.1%)	30 (1.7%)	136 (5.2%)	342 (11%)	
No	2014 (99.4%)	12,271 (97.9%)	1704 (98.3%)	3,518 (97.8%)	3,343 (92.8%)		1719 (98.5%)	11,896 (96.9%)	1754 (98.3%)	2,493 (94.8%)	2,768 (89%)	
Operation						<0.001						<0.001
Yes	124 (6.1%)	8,479 (67.6%)	1,291 (74.5%)	3,099 (86.2%)	1884 (52.3%)		225 (12.9%)	8,827 (71.9%)	1,407 (78.9%)	2,106 (80.1%)	1703 (54.8%)	
No	1903 (93.9%)	4,057 (32.4%)	442 (25.5%)	497 (13.9%)	1716 (47.6%)		1,520 (87.1%)	3,445 (28.1%)	377 (21.1%)	523 (19.9%)	1,407 (45.2%)	
Prognosis						<0.001						<0.001
Recovered/improved	1956 (96.5%)	11,946 (95.3%)	1,670 (96.4%)	3,513 (97.7%)	3,332 (92.6%)		1,685 (96.6%)	11,989 (97.7%)	1740 (97.5%)	2,552 (97.1%)	3,001 (96.5%)	
Not improved/died/others	71 (3.5%)	590 (4.7%)	63 (3.6%)	83 (2.3%)	268 (7.4%)		60 (3.4%)	283 (2.3%)	44 (2.5%)	77 (2.9%)	109 (3.5%)	
Readmission						<0.001						<0.001
Yes	22 (1.1%)	408 (3.3%)	38 (2.2%)	22 (0.6%)	219 (6.1%)		22 (1.3%)	130 (1.1%)	16 (0.9%)	31 (1.2%)	44 (1.4%)	
No	2005 (98.9%)	12,128 (96.7%)	1,695 (97.8%)	3,574 (99.4%)	3,385 (93.9%)		1723 (98.7%)	12,142 (98.9%)	1768 (99.1%)	2,598 (98.8%)	3,066 (98.6%)	
Length of hospital stay	9 (7–13)	8 (5–10)	6 (4–8)	3 (2–5)	10 (7–16)	<0.001	10 (7–15)	7 (5–9)	6 (4–8)	3 (2–5)	9 (6–15)	<0.001
Total hospital costs	8181.7 (5817.9–12031.7)	12253.3 (6596.1–16311.8)	7353.7 (5345.3–10,870)	4875.8 (3886.9–6012.2)	14912.9 (7862.3–29132.8)	<0.001	8951.3 (6323.5–13803.3)	11191.9 (6750.8–13840.6)	7771.6 (5873.1–10485.2)	4772.2 (3194.5–7242.7)	13272.8 (7795.6–27388.1)	**<0.001**

## 4 Discussion

This large-scale, retrospective study provides a comprehensive analysis of the characteristics and clinical outcomes of pediatric UI requiring hospitalization over a decade. Furthermore, it delineates the shifts in these epidemiological patterns during the COVID-19 pandemic.

The total number of pediatric hospitalizations due to UI during the 5 years following the COVID-19 pandemic showed a decline compared to the five-year period preceding it. Marked reductions were observed particularly in 2020 and 2022, with a gradual upward trend emerging from 2023 onward—patterns consistent with the evolving impacts of the pandemic. The first known case of COVID-19 was reported in December 2019. In response, China enforced a strict and comprehensive dynamic zero-COVID policy during the initial outbreak year of 2020. Similarly, in 2022, stringent public health measures were reinstated to combat the Omicron variant (17). Stay-at-home mandates and the transition to online education likely contributed to reduced outdoor and physical activity among children, thereby decreasing the incidence of UI (18). Conversely, during 2021, a period of relative stability and normalized social activity, the number of injury-related hospitalizations rose compared to both 2020 and 2022. By 2023, as China transitioned to routine COVID-19 management and children resumed more outdoor activities and in-person schooling, the rate of UI admissions began to climb again (17, 19).

Our data reaffirm that male children are consistently at higher risk for severe UI requiring hospitalization—a finding well-documented in global literature and often attributed to greater risk-taking behavior and increased exposure to hazardous environments among boys (20). Furthermore, the proportion of boys hospitalized due to UI gradually increases with age, a trend consistent with prior studies (12). This pattern may be explained by the fact that as children grow older, their capacity to engage in a wider range of activities expands, thereby increasing their exposure to potential risks. However, this expanding involvement is not matched by a sufficient development in their ability to recognize and avoid dangerous situations, which may contribute to the continued occurrence of accidents among boys (21). Consequently, older male children face a higher risk of fatal UI. Notably, the proportion of male children hospitalized due to UI has exhibited a discernible downward trend in recent years. This shift can be linked to several contributing factors: first, targeted interventions by both schools and families have effectively addressed boys’ natural inclinations toward high-energy and adventurous behaviors, resulting in measurable improvements. Second, there has been heightened parental emphasis on curbing excessive risk-taking among boys, fostering greater safety awareness. Additionally, during the COVID-19 pandemic, lockdowns and social restrictions led to significant shifts in children’s behavioral patterns. Traditional outdoor and high-risk activities—more prevalent among boys—were considerably reduced, thereby further lowering the incidence of UI in this group and contributing to the decline in hospitalizations. Amid these developments, it remains crucial not to neglect the prevention of UI among female children.

According to our data, children in rural areas face a significantly higher risk of UI compared to those in urban settings. This observation aligns with existing studies conducted in China (22), India (23) and Ireland (24). Rural environments are often characterized by a higher presence of natural hazards—such as open water, farmland, and uneven terrain—coupled with a prevalent “free-range” parenting approach and a larger proportion of left-behind children. These factors contribute to reduced adult supervision and diminished safety awareness, collectively elevating the risk of injuries among rural children. Prior to the COVID-19 pandemic, the proportion of rural children among those hospitalized for UI had been declining year by year, indicating initial success in the prevention and management of such injuries in rural areas. However, studies revealed that during the 2020–2022 global COVID-19 outbreak, the share of hospitalizations due to UI among rural children increased. This shift may be attributed to stricter containment policies implemented in urban settings, whereas children in rural areas retained relatively greater freedom of movement, leading to higher exposure to accident risks. By 2023, as social activities resumed normality, the proportion of urban children hospitalized for UI rose markedly, reflecting heightened risks associated with expanded daily mobility, increased traffic participation, and more frequent outdoor recreational activities among this group.

Based on age distribution, toddlers exhibited the highest rate of hospitalization due to UI, followed by school-aged children, preschoolers, infants, and adolescents. This pattern aligns with the majority of existing literature (22). The elevated injury incidence among toddlers can be ascribed to their high activity levels, curiosity, underdeveloped safety awareness, and potential lapses in parental supervision. The study observed a decline in injury-related hospitalizations among infants and toddlers following the COVID-19 pandemic, likely attributable to increased remote work opportunities for parents, which facilitated greater supervision and reduced the incidence of severe injuries. In contrast, UI among school-aged children and adolescents rose during the same period. With guardians often distracted by work or domestic tasks, these older children spent more time unsupervised at home. Despite being perceived as more capable of self-care, they were in fact more likely to engage in high-risk activities—such as rough play or improper use of tools—resulting in a higher injury rate.

Regarding injury types, the most common causes of hospitalization were fall/slip, traffic accidents, foreign body, thermal and blunt injuries. Fall/slip consistently ranked as the leading cause across all age groups, a finding consistent with previous studies which identified unintentional falls as the predominant cause of injury among children of all age (12, 25). According to the U.S Centers for Disease Control and Prevention (CDC), falls were the leading cause of nonfatal injury for children <15 years old (26). Post-pandemic, hospitalizations due to traffic accidents, foreign body, and thermal injury decreased—a trend likely linked to COVID-19 restrictions such as lockdowns, school closures, and stay-at-home orders, which reduced outdoor exposure, commuting, and traffic-related risks (27). Similarly, improved parental oversight and fewer meals eaten away from home may have contributed to the reduction in foreign body and thermal injury incident (28). However, prolonged time spent indoors also appears to have increased the incidence of falls severe enough to require medical attention. This is supported by data showing that accidental falls occur predominantly in domestic settings: 92.1% among children under 5 years old and 71% among those aged 5 to 13. Moreover, the vast majority of these incidents (98.1%) are unintentional, and most (74.9%) are the result of falls from ground level (29). confined living spaces containing hazards such as stairs and sharp furniture edges likely elevated the risk of falls and blunt injuries (e.g., collisions with furniture), leading to a higher share of hospitalizations from these causes. Fall/slip represent the most common cause of hospitalization from UI across all age groups, with a steadily increasing incidence year over year. This trend highlights the need for parents to not only implement household safety modifications but also to apply differentiated supervision approaches. While close monitoring remains crucial for infants and toddlers, it is equally important to provide tailored safety education to school-aged children and adolescents.

Moreover, research has documented a notable surge in unintentional poisoning incidents among adolescents following the COVID-19 pandemic. A specific study revealed that the majority of such poisonings occurred in youths between the ages of 13 and 18 (30). During the pandemic, pediatric emergency department visits related to poisoning rose significantly, with adolescent cases increasing by 57.5%. Notably, intentional self-poisoning accounted for 53.4% of all poisoning events in this age group (31). Extended periods of social isolation have been shown to adversely affect the mental well-being of children and adolescents, amplifying psychological distress and promoting risky behaviors. Adolescence is a critical developmental period marked by the transition into societal adulthood—a phase during which peer interaction and a sense of belonging play vital roles (32). During severe public health crises, the disruption of daily routines and social networks can elevate the risk of self-poisoning among psychologically vulnerable adolescents, underscoring the urgent need for increased awareness and intervention from both families and society.

The analysis of injury anatomy offers a clear correlation between developmental stage and injury pattern. The high prevalence of head injuries in younger children is a consistent and concerning feature, attributable to their proportionally larger head size, higher center of gravity, and underdeveloped motor skills and coordination (33). As children grow and engage in more complex physical activities like sports and cycling, injuries to the extremities become more common, as seen in school-aged children and adolescents.

A critical finding of this study is the contrasting trends in healthcare utilization. While the need for ICU care and surgical intervention showed an increasing trend, overall hospital outcomes improved, as evidenced by higher recovery/improvement rates, lower readmission, and shorter lengths of stay. This paradox may be explained by advances in pediatric trauma care, more efficient clinical management protocols, and possibly the admission of less severely injured patients during the pandemic. Finally, the analysis of clinical outcomes by injury type provides valuable insights for resource allocation and clinical preparedness. Traffic accidents, associated with the poorest outcomes (highest ICU rate, lowest recovery rate, highest cost), represent the most burdensome type of injury, emphasizing the paramount importance of road safety measures. The CDC has reported traffic accidents as the leading cause of injury and/or deaths for children older than 4 years (26). The WHO estimated that traffic injuries were the leading cause of fatal injuries among children worldwide (34). More than 18,500 children are killed annually in traffic accidents in China, with mortality rate at 2.5 times of the Europe and 2.6 times of the United States (35). In contrast, foreign body injuries, while requiring frequent surgical intervention, resulted in the shortest stays and lowest costs, suggesting that these are often managed effectively with standardized procedures.

### 4.1 Limitations and implications

This study has several limitations: First, the single-center design may have introduced selection bias and limits the generalizability of our results to other populations or healthcare settings. Second, although dividing the study period into pre-pandemic (2015–2019) and pandemic (2020–2024) phases was necessary to detect overarching trends, this binary classification does not fully capture the complex and multi-phased nature of the COVID-19 pandemic. Specifically, it fails to account for the substantial heterogeneity within the 2020–2024 period, including distinct phases such as strict lockdowns, the dynamic zero-COVID strategy, the Omicron wave, and the subsequent transition to routine pandemic management. Moreover, this approach could not exclude the influence of several confounding factors when comparing injury patterns before and after the pandemic onset. These include changes in healthcare-seeking behavior (e.g., avoidance of hospitals during the pandemic), declining birth rates and shifts in referral patterns (e.g., only severe cases presenting to tertiary hospital). Such factors may have independently influenced the observed trends and should be carefully considered in future research. Therefore, further studies employing more granular methodologies, such as the use of comprehensive injury surveillance data, are warranted to better elucidate these complex dynamics. Despite these limitations, the findings have clear implications. The post-COVID-19 changes indicate that injury patterns are not static and can shift with major societal changes. Prevention strategies must therefore be dual-focused: maintaining efforts against traditional hazards like traffic accidents and falls, while also adapting to new realities, such as the increased time children spend in domestic settings. Targeted education for parents and caregivers on preventing home-based injuries, especially for infants and toddlers, is crucial.

## 5 Conclusion

In conclusion, this study delineates the evolving landscape of pediatric UI before and after the COVID-19 pandemic. While the fundamental causes of injury remain, their proportions and severities have shifted, influenced by changes in children’s living environments and activities. These results underscore the importance of continuous epidemiological surveillance to inform and refine effective, age-specific prevention strategies and to optimize clinical resource allocation for pediatric injury care.

## Data Availability

The datasets generated and/or analyzed during the current study are not publicly available due to privacy and ethical restrictions of retrospective clinical data, but are available from the corresponding author upon reasonable request.
